# Low molecular weight glutenin subunit gene *Glu-B3h* confers superior dough strength and breadmaking quality in wheat (*Triticum aestivum* L.)

**DOI:** 10.1038/srep27182

**Published:** 2016-06-07

**Authors:** Yaping Wang, Shoumin Zhen, Nana Luo, Caixia Han, Xiaobing Lu, Xiaohui Li, Xianchun Xia, Zhonghu He, Yueming Yan

**Affiliations:** 1 College of Life Science, Capital Normal University, Beijing 100048, China; 2 Institute of Crop Science, Chinese Academy of Agricultural Sciences (CAAS), Beijing 100081, China

## Abstract

Low molecular weight glutenin subunit is one of the important quality elements in wheat (*Triticum aestivum* L.). Although considerable allelic variation has been identified, the functional properties of individual alleles at *Glu-3* loci are less studied. In this work, we performed the first comprehensive study on the molecular characteristics and functional properties of the *Glu-B3h* gene using the wheat cultivar CB037B and its *Glu-B3* deletion line CB037C. The results showed that the *Glu-B3h* deletion had no significant effects on plant morphological or yield traits, but resulted in a clear reduction in protein body number and size and main quality parameters, including inferior mixing property, dough strength, loaf volume, and score. Molecular characterization showed that the *Glu-B3h* gene consists of 1179 bp, and its encoded B-subunit has a longer repetitive domain and an increased number of α-helices, as well as higher expression, which could contribute to superior flour quality. The SNP-based allele-specific PCR markers designed for the *Glu-B3h* gene were developed and validated with bread wheat holding various alleles at *Glu-B3* locus, which could effectively distinguish the *Glu-B3h* gene from others at the *Glu-B3* locus, and have potential applications for wheat quality improvement through marker-assisted selection.

Wheat (*Triticum aestivum* L.), one of the three major cereal crops in the world, is a critical source of energy and nutrients in the human diet and has excellent processing characteristics. Wheat dough is used to make various food products including bread, noodles, cakes, and biscuits^1^. The seed storage proteins in wheat consist of monomeric gliadins and polymeric glutenins that determine the extensibility and elasticity of dough, respectively^2^,^3^. According to their mobility, as determined by sodium dodecyl sulfate polyacrylamide gel electrophoresis (SDS-PAGE), polymeric glutenins are subdivided into high and low molecular weight glutenin subunits (HMW-GS and LMW-GS, respectively), of which, LMW-GS accounts for ~60% of the glutenins and primarily determines dough strength and viscosity, thus playing a significant role in flour processing quality^4^,^5^. Some studies have shown that the effects of LMW-GS on both dough resistance and dough extensibility are more favorable than the effects of HMW-GS^6^,^7^.

LMW-GS is encoded by *Glu-A3, Glu-B3*, and *Glu-D3* loci on the short arms of chromosomes 1 A, 1B, and 1D, respectively, and these loci are linked to the *Gli-1* locus, encoding gliadins^2^,^8^. The molecular structure of LMW-GS contains four typical regions: (1) a signal peptide containing 20 amino acids removed in the maturation process, (2) a short N-terminal region with 13 amino acids containing one cysteine, (3) a repetitive domain rich with glutamine containing 70–186 amino acids as the variable region of gene size, and (4) a C-terminus rich with cysteine and glutamine. The C-terminus has three regions: (1) a cysteine-rich structure containing five cysteines, (2) a domain rich with glutamine, with only one cysteine and some tandem glutamines, and (3) a conserved region of the C-terminus with the last cysteine 9,10,11.

According to the molecular weight of the subunits, LMW-GS could be classified into B, C, and D type subunits, of which, B type subunits are the main type of LMW-GS and belong to alkaline proteins. The isoelectric point of the C type subunits varies from weakly acidic to strongly basic, while the D type subunits possess a low percentage of total LMW-GS and are only expressed in certain cultivars^8^,^12^. LMW-GS is classified into three subclasses (LMW-m, LMW-s, and LMW-i) based on their N-terminal amino acid sequences, in which m, s, and i represent methionine, serine, and isoleucine, respectively, the first amino acid residues of the mature proteins^13^,^14^. The N-terminus of LMW-m contains METSHIPGL-, METSRIPGL-, and METSCIPGL-. The N-terminus of LMW-s is SHPGL-, while the LMW-i type lacks the typical N-terminal sequence, following the signal peptide is the repetitive domain ISQQQQ^13^,^15^,^16^.

The genes encoding LMW-GS do not contain an intron, which was estimated to have 10–15 or 30–40 copies in hexaploid wheat^10^,^17^ due to extensive allelic variation present at *Glu-3* loci. Gupta and Shepherd^18^ identified and named 20 alleles at *Glu-3* in hexaploid wheat, including six at *Glu-A3*, nine at *Glu-B3,* and five at *Glu-D3*. These alleles were shown to have differential effects on wheat processing quality. Ikeda *et al*.^19^ isolated several LMW-GS genes and classified them into 12 groups in a soft wheat cultivar. Dong *et al*.^1^ also identified four, three, and seven LMW-GS genes at the *Glu-A3, Glu-B3*, and *Glu-D3* loci, respectively, in Xiaoyan 54.

To date, studies on the functional properties of individual alleles are still challenging due to the lack of LMW-GS mutants at the *Glu-3* locus and the difficulty of wheat genetic transformation. Previous studies were performed mainly using near-isogenic lines (NILs) to understand the relative importance of different *Glu-3* alleles for wheat quality. The effects of different *Glu-3* loci on processing qualities have been ranked in bread wheat. *Glu-A3* and *Glu-B3* alleles are supposedly more important than *Glu-D3* alleles in terms of wheat processing qualities. Zhang *et al*.^20^ studied the function of 18 LMW-GS alleles in bread wheat using Aroona NILs, and found that *Glu-A3e* exhibited the worst performance with respect to almost all quality properties. *Glu-B3b, Glu-B3g,* and *Glu-B3i* were more highly correlated with superior breadmaking quality than the other *Glu-B3* alleles, whereas the *Glu-D3* alleles showed no significant effects on bread wheat quality. Jin *et al*.^21^ used a set of Aroona NILs of bread wheat to clarify the contribution of each allele to processing quality, and showed that alleles *Glu-A3b, Glu-A3d, Glu-B3g*, and *Glu-B3f* make significant contributions to mixograph properties. More recently, Zhen *et al*.^22^ used a natural mutation at the *Glu-A3* locus to study the function of the *Glu-A3a* allele in bread wheat, and found that *Glu-A3a* significantly affects dough strength and breadmaking quality. However, functional studies on most other *Glu-3* alleles have not been performed.

In the current study, we used the *Glu-B3* deletion line, which was derived from mutation screening during immature embryo culture for spring wheat CB037B (*Triticum aestivum* L., 2n = 6× = 42, AABBDD) genetic transformation research in our laborator, to conduct the first comprehensive study on the molecular characteristics and functional properties of the LMW-GS allele *Glu-B3h* using proteomic and molecular biology approaches. SDS-PAGE, Two-dimensional electrophoresis (2-DE), reversed-phase ultra performance liquid chromatography (RP-UPLC) and liquid chromatography-tandem mass spectrometry (LC-MS/MS) were used to separate and characterize the *Glu-B3h* encoded B-subunit, sequence-tagged site polymerase chain reaction (STS-PCR) were used to confirm the deletion of *Glu-B3h* in CB037C and allele-specific PCR (AS-PCR) is to amplify the *Glu-B3h* gene in CB037B. Light microscopy observation and scanning electron microscope (SEM) were designed to analysis the protein body (PB) number and size, the function properties of LMW-GS Glu-B3h were measured by Mixgraph and Extenograph, a variety of bioinformatics method including sequence alignment, secondary structures prediction and phylogenetic analysis were performed to understand the molecular characteristic of *Glu-B3h*, considering the superior processing properties of *Glu-B3h* encoded LMW-GS, the single nucleotide polymorphism (SNP)-based molecular markers for the *Glu-B3h* gene were developed and validated. Our results demonstrate that *Glu-B3h* has important effects on dough strength and breadmaking quality and reveal its potential value for contributing to wheat quality improvement.

## Results

### Agronomic traits and yield performance of CB037B and CB037C at three locations

The results from three locations (Beijing, Yinchuan, and Xining) showed that CB037B and CB037C have relatively high purity and genetic stability (Supplementary Fig. S1 and Table S1). Plant morphological characteristics, main agronomic traits, and yield performance of both cultivars, including plant and spike morphology, grain weight, and yield, were not significantly different (Supplementary Table S1). Thus, both cultivars were highly consistent without regarding growth and development at the different locations. However, the CB037B developed in Beijing, Yinchuan and Xining showed significant difference in agronomic traits and yield performance, and the same trend also appeared in the CB037C which were planted in these three locations, which demonstrating that environmental factors have significant effects on agronomic traits and yield performance.

### Separation and characterization of the *Glu-B3h* encoded B-subunit

**Figure 1:**
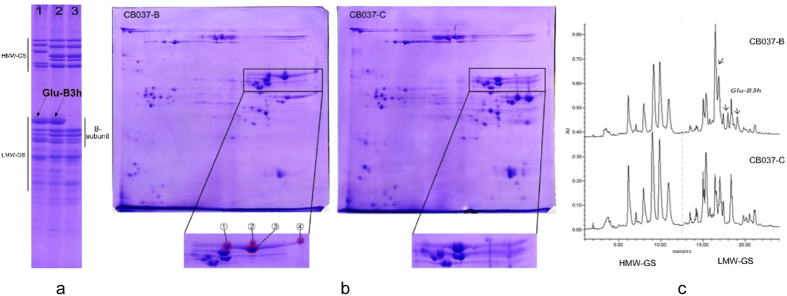
Identification of Glu-B3h from CB037B and *Glu-B3h* deletion line CB037C by SDS-PAGE (a), 2-DE (b) and RP-UPLC (c). **(a)** The *Glu-B3h* encoded B-subunit as well as LMW-GS and HMW-GS were indicated. 1. Aroona B3h; 2. CB037B; 3. CB037C.**(b)** Four protein spots differentially expressed between CB037B and CB037C were marked by 1, 2, 3 and 4. **(c)** Three protein peaks encoded by *Glu-B3h* as well as glutenin subunits in CB037B and CB037C were indicated.

The glutenin composition of CB037B and CB037C was separated and identified by SDS-PAGE (Fig. 1a). Both cultivars had the same HMW-GS composition at the *Glu-1* locus (1Ax1, 1Bx17 + 1By18, 1Dx5 + 1Dy10). At the *Glu-3* locus, CB037B had *Glu-A3a, Glu-B3h*, and *Glu-D3d*. CB037C had the same *Glu-3* composition as CB037B, but *Glu-B3h* encoding one abundant LMW-B subunit was absent. 2-DE analysis revealed that *Glu-B3h* encodes four protein components (spots 1, 2, 3 and 4 in Fig. 1b). RP-UPLC further confirmed the absence of one abundant LMW B-subunit encoded by *Glu-B3h* in CB037C, which eluted after 16.44 min (Fig. 1c). The B-subunit peak had the highest expression and accounted for 16.6% of the total glutenin protein content and 30.8% of the total LMW-GS content in CB037B.

The *Glu-B3h* encoded protein band on the SDS-PAGE gel (Fig. 1a) and four protein spots on the 2-DE gel (spots 1, 2, 3 and 4 in Fig. 1b) were collected and digested by trypsin, and then analyzed by LC-MS/MS. Based on these results, four peptide sequences (13.52% coverage) were matched with the *GluB3-3* gene (AC number EU369717), encoding the LMW B-subunit in the *T. aestivum* L. cultivar Aroona-B3h, of which “K.VFLQQQCSPVAM*PQSLAR.S” was completely consistent with the 236–255 amino acid sequence of the *GluB3-3* coding protein (Supplementary Table S2). Thus, the deleted B-subunit in CB037C was determined to be encoded by *Glu-B3h*.

### Confirmation of *Glu-B3h* locus deletion in CB037C by STS-PCR markers

STS-PCR was used to confirm whether the *Glu-B3* locus was absent or silent in CB037C. The *Glu-B3h* gene at the *Glu-B3* locus in CB037B and CB037C was amplified using a pair of STS primers. One clear fragment of 1022 bp was amplified in CB037B and Aroona-B3h with the *Glu-B3h* gene, but no products were obtained from CB037C or other cultivars lacking the *Glu-B3h* encoded B-subunit (Supplementary Fig. S2). One amplified 1022 bp fragment was collected and sequenced, and the results indicated that it was identical to the sequence from 114 to 1136 bp of the *Glu-B3h* gene (Supplementary Fig. S3). These results confirm that the *Glu-B3* locus was deleted in CB037C.

### Protein body comparison of CB037B and CB037C during grain development

**Figure 2:**
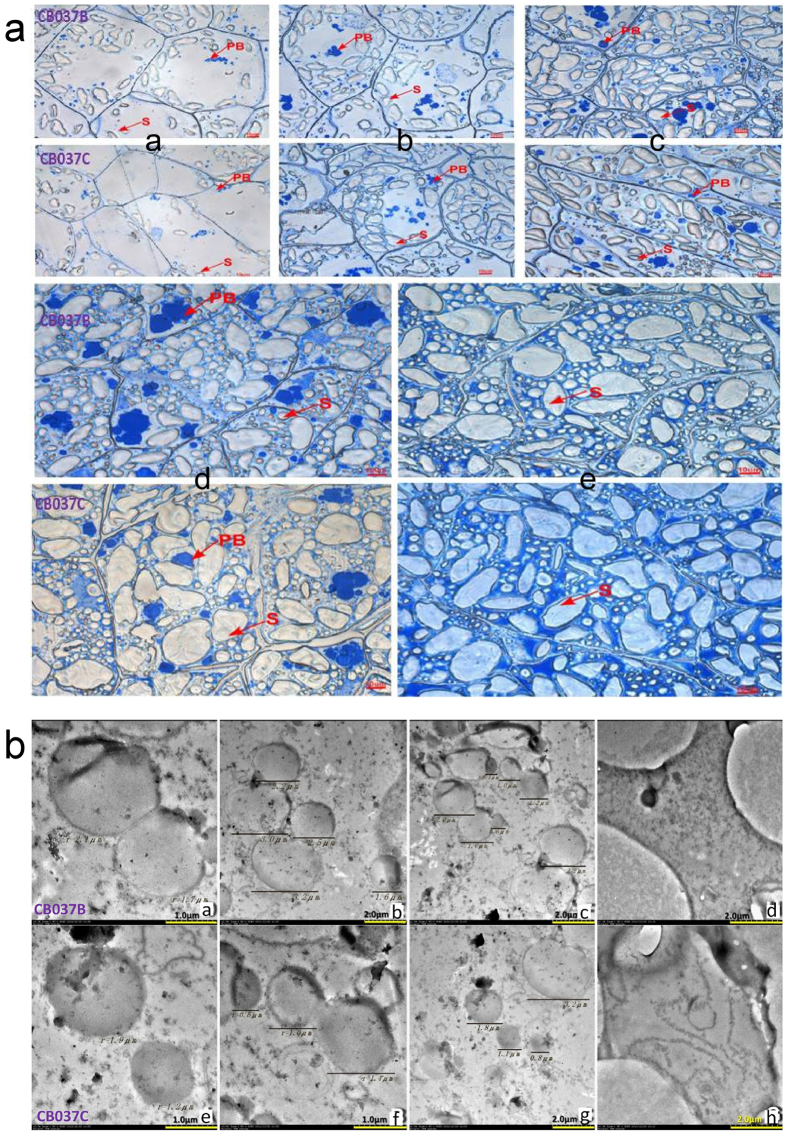
Developmental changes of PB during grain development of CB037B and CB037C. **(a)** Light microscopy observation of commassie blue staining on sections of protein bodies at 8, 11, 14, 17, 26 DPA. Numbers a, b, c, d and e correspond to the 8, 11, 14, 17 and 26 DPA; PB: protein bodies; S: starch; Bars: 10 μm. **(b)** Scanning electron microscopy observation of dynamic changes about endosperm structures from 4-day intervals after flowering during grain development.

Seed storage proteins are synthesized in the endoplasmic reticulum and are transferred and accumulate in the PB after being processed in the Golgi apparatus. PB differences between CB037B and CB037C during grain development were observed by different microscopy techniques (Fig. 2). Light microscopy observation of transverse slices of the grain endosperm showed that PB formation and developmental features were similar in both cultivars, but the number and sizes of PBs were significantly different (Fig. 2a). At 8 days post anthesis (DPA), starch granules were clearly visible and a handful of PBs were formed, while at 11–17 DPA, the number and size of the PBs increased quickly and some gathered PB fusion for glutenin macropolymers (GMP) formation. The PBs were fully combined to form the uniform protein matrix at 26 DPA in both cultivars. However, deletion of the *Glu-B3h* encoded B-subunit resulted in a reduction in PB number and size. SEM further verified that CB037B had more and larger PBs than CB037C (Fig. 2b), consistent with the results from light microscopy (Fig. 2a).

### Functional properties of the *Glu-B3h* gene

**Figure 3:**
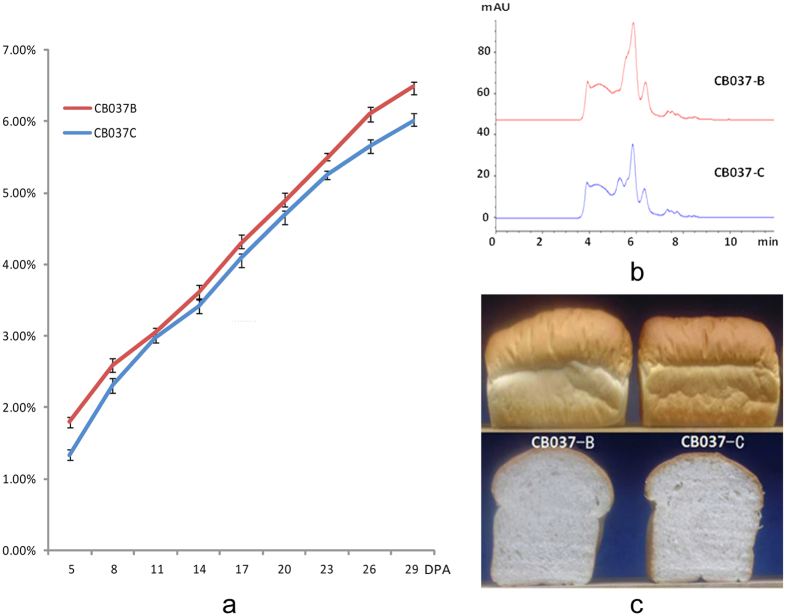
The comparison of GMP contents and pan bread between CB037B and CB037C. (**a**) GMP content determination of CB037B and CB037C at 5, 8, 11, 14, 17, 20, 23, 26, 29 DPA. **(b)** Separation and identification of GMP in the CB037B and CB037C by SE-HPLC. (**c**) Pan bread appearance of CB037B and CB037C.

Comparison of the two cultivars for their main quality traits over three locations showed that the deletion of LMW-GS *Glu-B3h* significantly reduced gluten strength and breadmaking quality (Table 1). In general, total grain protein contents of CB037B and CB037C were not significantly different, but GMP contents during grain development were significantly reduced in CB037C (Fig. 3a,b).

Main Mixgraph and Extenograph parameters reflecting dough kneading resistance were also significantly lower in CB037C than in CB037B. These parameters included tolerance index, development time, stability, max resistance, and extensibility. Finally, the reduction of these mixing properties led to a significant reduction in loaf volume and score (Fig. 3c; Table 1). These results were highly consistent over the three locations. In addition, the locations had greater differences for quality parameters, indicating the environmental effects on gluten quality formation.

### Molecular characterization of the *Glu-B3h* gene

According to the results from tandem mass spectrometry and coding sequences of *GluB3-3*, a pair of AS-PCR primers, LB3F and LB3R, was designed and synthesized, and used to amplify the *Glu-B3h* gene from CB037B. One clearly amplified band of approximately 1300 bp was present in CB037B (Supplementary Fig. S4). After collecting, cloning, and sequencing, a complete 1179 bp ORF was obtained, corresponding to the size typical of LMW-GS genes that range from 900 bp to 1200 bp. Sequence alignment showed that the amplified sequences had the basic structural characteristics of LMW-GS genes and no internal stop codons. After blasting this gene in NCBI, we found that the cloned nucleotide sequence was completely consistent with the *GluB3-3* gene from the wheat cultivar Aroona-B3h (AC number EU369717), therefore, it was determined to be *Glu-B3h* gene.

The deduced N-terminal sequence of the *Glu-B3h* gene was MENSHIPGL-. Since the peptide MEN was likely to be removed from the original protein^23^, the first amino acid of the mature protein of *Glu-B3h* was serine, indicating that this protein belongs to the LMW-s type subunit. The complete coding sequence of *Glu-B3h* was aligned with 20 known LMW-s genes from wheat to detect SNPs and insertions/deletions (InDels) variation, indicating that *Glu-B3h* had six SNPs at different positions. Two of the SNPs were at 972 bp and 1110 bp, and four were at 133 bp, 174 bp, 1117 bp, and 1172 bp, belonging to synonymous and nonsynonymous SNPs (Table 2). In particular, *Glu-B3h* had a long insertion fragment of 42 bp at position 464–505, making it longer than other LMW-GS genes.

The deduced amino acid sequences of the *Glu-B3h* gene included four clear regions of the typical primary structure of LMW-GS (Supplementary Fig. S5). The repetitive domain of *Glu-B3h* contained 15 hexapeptides (consensus PPFSQQ and QQPVLP) and two nonapeptides (QQPSFSQQQ), which accounted for 57% of the repetitive domain and were mainly responsible for the length expansion and general hydrophilic characteristics of LMW-GS.

### Secondary structure analysis of the Glu-B3h encoded protein

Secondary structures of the deduced mature glutenin subunits of *Glu-B3h* and five other LMW-GS from bread wheat (AY542896, AY831886, AY724436, FJ824789, and JX877832) were predicted on the PSIPRED server, and a comparative analysis was performed (Table 3). The results showed that the α-helices and β-strands were relatively conserved in repetitive domain. C-terminal and dispersed in a normal configuration in repetitive domain. The *Glu-B3h* encoded B-subunit (AC number EU369717) contained four α-helices located mainly at C-terminal I, and one β-strand in the conserved C-terminal region. Comparative analysis showed that the number of α-helices in the *Glu-B3h* encoded protein was much higher than AY542896, which was confirmed to have positive effects on wheat bread quality^24^. These results indicate that the *Glu-B3h* encoded subunit could be associated with superior gluten quality and may be responsible for the significant reduction in dough strength and breadmaking quality of CB037C.

### Phylogenetic analysis among *Glu-3* genes

**Figure 4:**
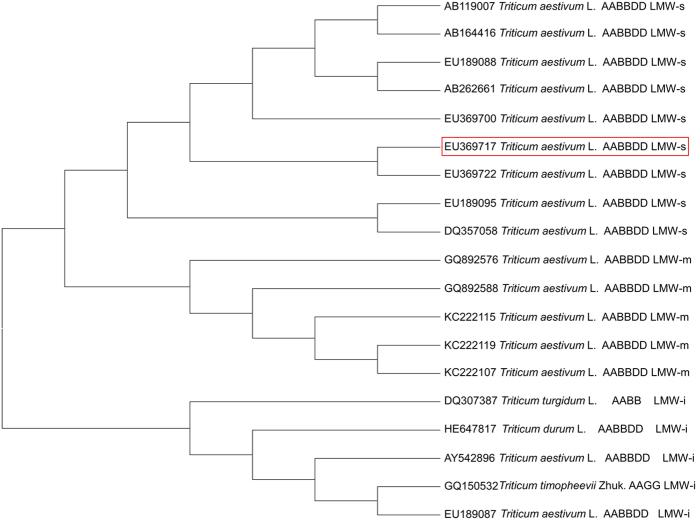
Phylogenetic tree of 19 LMW-GS constructed by the complete coding DNA sequences. 19 LMW-GS genes named EU369717, AB119007, AB164416, EU189088, AB262661, EU369700, EU369722, EU189095, DQ357058, GQ892576, GQ892588, KC222115, KC222119, KC222107, DQ307387, HE647817, AY542896, GQ150532 and EU189087.

To understand the phylogenetic relationship among the LMW-GS genes at the *Glu-3* locus, the coding sequences of the *Glu-B3h* gene and an additional 18 LMW-GS genes from wheat and related species were used to construct a homology tree using MEGA6 software. This tree included eight LMW-s (AB119007, AB164416, EU189088, AB262661, EU369700, EU369722, EU189095, and DQ357058), five LMW-m (GQ892576, GQ892588, KC222115, KC222119, and KC222107), and five LMW-i (DQ307387, HE647817, AY542896, GQ150532, and EU189087) type genes from *T. aestivum, T. durum, T. turgidum*, and *T. timopheevii*. These results indicate that LMW-i type genes underwent greater divergence during evolution and were clustered into a separate clade, while LMW-m and LMW-s type genes were separated into another clade in which two subgroups were present. *Glu-B3h* showed a close relationship with other LMW-s genes, particularly with EU369722 from bread wheat (Fig. 4).

The divergence timing of *Glu-B3h* and an additional 11 LMW-GS genes were calculated using MEGA6.0 software to further investigate the evolutionary relationships among the LMW-GS genes. LMW-s was more related to LMW-m than LMW-i, their divergence occurred around 6–8 million years ago (MYA; Supplementary Table S3). LMW-i type genes diverged much earlier, at about 11–14 MYA, suggesting that LMW-i type genes are the most original in the LMW-GS family.

### Development and validation of SNP-based molecular markers for the *Glu-B3h* gene

**Figure 5:**
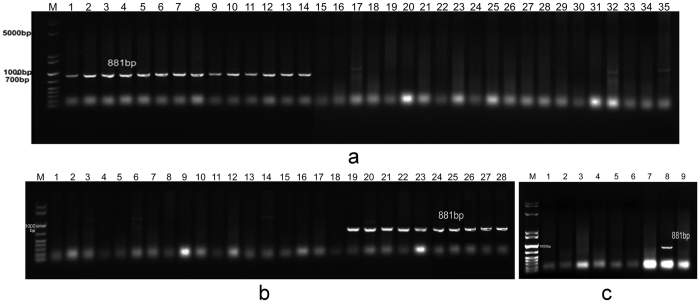
Development and validation of SNP-based molecular marker for Glu-B3h. (**a**) PCR amplification from different wheat cultivars: 1. CB037B. 2–35 corresponds to 26–59 in Table S4. **(b)** PCR amplification from RILs from CB037B × Ningchun 4 (1–9, 18–22) and CS-1S^l^(1B) × CB037B (10–17, 23–27). 28. CB037B. **(c)** PCR amplification from Aroona NILs. 1. Aril20-1, 2. Aril21-2. 3. Aril23-4. 4. Aril24-3. 5. Aril26-1. 6. Aril27-6. 7. Aril29-4. 8. Aril28-4. 9. Aril30-1. M is marker (10 kb, 8 kb, 5 kb, 2 kb, 1.6 kb, 1 kb, 700 bp, 500 bp, 400 bp, 300 bp, 200 bp, 100 bp).

Based on the SNPs detected in the *Glu-B3* gene, a pair of primers specific for *Glu-B3h (Glu-B3h* F: CCACCACAACAAACATTAA, *Glu-B3h* R: TGCCCGAGTTG CTGTTGT) was designed and tested in wheat cultivars with various allelic compositions at the *Glu-B3* locus, as identified by SDS-PAGE (Supplementary Table S4 and Fig. S6). PCR amplification showed that a fragment of 881 bp was present in cultivars with the *Glu-B3h* gene, while no products were obtained from the other wheat genotypes with other *Glu-B3* alleles (Fig. 5). The SNP-based molecular markers developed were further validated using different cultivars (Fig. 5a), two F_2_ populations from CB037B × Ningchun 4 and CS-1 S^l^/1B × CB037B, two recombinant inbred lines (RILs) from CB037B × Ningchun 4 and CS-1S^l^/1B × CB037B (Fig. 5b), and eight Aroona NILs (Fig. 5c). These molecular markers have potential applications for wheat quality improvement through marker-assisted selection.

## Discussion

In this study, we used the *Glu-B3* deletion mutant CB037C to investigate the molecular characteristics and functional properties of the *Glu-B3h* gene. Our results provide new insights into the allelic variation mechanism and molecular basis of gluten quality formation. The *Glu-B3h* gene, with specific structural features, showed potential value for wheat gluten quality improvement through use of SNP-based molecular markers. Here, we focused on several key issues pertaining to the *Glu-B3h* gene.

Our results show that *Glu-B3h* encodes an LMW-s type B-subunit and has close phylogenetic relationships with other LMW-s genes. Both LMW-m and LMW-s possess similar structures and close evolutionary relationships at the amino acid sequence level^25^. Thus, the LMW-s type gene is considered a derivative of LMW-m type subunits, and its divergence occurred ~7.81 MYA^26^. In the present study, the divergence time estimation for the three types of LMW-GS genes showed that LMW-s and LMW-m genes diverged at about 6–8 MYA (Supplementary Table S3), which is consistent with the findings by Li *et al*.^26^. The divergence time of LMW-m and LMW-i was about 12~14 MYA, which is consistent with that described by Wang *et al*.^23^. It is known that the primary genetic mechanisms for allelic variation at the *Glu-1* and *Glu-3/Gli-2* loci mainly involve point mutations, unequal crossing-over, slip-mismatching, and intra-chromosomal illegitimate recombination^8^,^26^,^27^. The extensive allelic variation of storage proteins mainly results from SNPs and InDels. In particular, *Glu-B3h* has six SNPs and a long insertion of 464–505 residues, which has led to a longer repetitive domain and larger gene size (Table 2). Considering the genome expansion and contraction from unequal crossing-over and illegitimate recombination^26^,^28^, it is likely that the *Glu-B3h* gene originated from unequal crossing-over or an illegitimate recombination event that may have occurred at 6.4 ~ 6.8 MYA. SNP variation gradually accumulated during the evolutionary process.

It is known that both expression levels and structural features of glutenin subunits are closely related to gluten quality^29^. The over-expressed HMW-GS 1Bx7^OE^ was confirmed to have positive effects on dough strength^30^. LMW-GS accounted for approximately 60% of the total protein in mature seeds^4^. LMW B-subunits are the most abundant among the LMW-GS in mature grains and have the greatest impact on wheat processing qualities^8^. Our results from RP-UPLC indicate that the *Glu-B3h* encoding subunit has an abundant protein peak, and its content accounts for 30.8% of the total LMW-GS in CB037B (Fig. 2c). Thus, the deletion of *Glu-B3h* led to a significant decrease in GMP content, dough strength, and breadmaking quality (Table 1; Fig. 5). LMW-GS participates in the formation of gluten macropolymers through intra- and inter-molecular disulfide bonds. Thus, disulfide bonds play important roles in determining the structure and properties of gluten proteins^31^. The different amounts and distribution of cysteine residues are closely related to the formation of secondary protein structure and dough quality, in which the first and seventh cysteine residues participate in forming the intermolecular disulfide bond, while the remaining residues are involved in the formation of intra-molecular disulfide bonds^13^. Helix-helix interactions could guide the formation of the intra-molecular disulfide bonds, thus, more α-helices might contribute to superior dough quality^32^. A previous study confirmed that AY542896 has positive effects on dough quality in bread wheat^14^. The secondary structure of *Glu-B3h* contains four α-helices, which was much higher than the one α-helix of AY542896 (Table 3), and may contribute to superior dough strength and breadmaking quality (Table 1).

The size and structural features of repetitive domains also have important effects on gluten quality. In general, LMW-GS genes are 900–1200 bp in length^10^ and contain a long repetitive domain that facilitates the formation of more α-helices and β-strands, and confers superior gluten structure and breadmaking quality^32^,^33^. Our results showed that *Glu-B3h* contains 1179 bp with a large insert fragment at nucleotides 464–505 that represent 14 amino acids (Table 2), which is longer than most other LMW-GS genes. In addition, the repetitive domain of *Glu-B3h* contains 15 hexapeptides (consensus PPFSQQ and QQPVLP) and 2 nonapeptides (QQPSFSQQQ), which account for 57% of the repetitive domain that is mainly responsible for the length of LMW-GS and contributes to superior dough quality^34^. These structural features may thus contribute to superior gluten quality. A recent report showed that the introduction of *Glu-B3h* into the cultivar Yumechikara made it possible to breed cultivars with good gluten and breadmaking quality^35^. This further confirms that *Glu-B3h* has potential applications for improving wheat gluten quality.

As a traditional method widely used for LMW-GS identification, SDS-PAGE has disadvantages in distinguishing some subunits with similar mobilities. It is also time-consuming and requires the use of toxic reagents. However, marker-assisted selection has become more effective for screening for superior genes. The development of molecular markers for the *Glu-3* locus is important for improving wheat quality. Wang *et al*.^36^ developed 10 allele-specific PCR markers based on SNPs and used them to discriminate the *Glu-B3* subunits. Based on sequence alignment of 13 LMW-GS genes previously identified, Zhang *et al*.^37^ developed a new molecular marker system to identify the LMW-GS gene family. Several STS markers for *Glu-3* subunits were developed and identified in more than 100 wheat cultivars^38^,^39^,^40^. Ikeda *et al*.^41^ developed 12 markers to differentiate 12 groups of LMW-GS genes of Norin 61. However, although considerable work on gene cloning and marker discovery has been performed, the superior LMW-GS genes and effective molecular markers widely used for wheat quality improvement are still limited. This may have resulted from the challenge of functional studies of the *Glu-3* gene and its highly repetitive sequences affecting the development and application of effective molecular markers.

SNPs are considered to be effective third-generation molecular markers that are powerful tools in marker-assisted breeding^42^. PCR-based molecular markers are generally fast to use, cost-efficient, and are subjected to few restrictions, which could provide a powerful tool for high-throughput selection during marker-assisted selection for wheat quality. In the present study, we developed SNP-based AS-PCR markers for the *Glu-B3h* gene and validated them in different wheat cultivars, F_2_ populations, NILs, and RILs (Supplementary Table S4; Fig. 5). By using these markers, breeders can now efficiently select this desirable subunit in early generations of a wheat quality breeding program.

## Materials and Methods

### Plant materials and field trials

Plant materials used in this study included CB037B (*Triticum aestivum* L., 2n = 6× = 42, AABBDD) and its *Glu-B3h* deletion line CB037C derived from mutation screening during immature embryo culture for wheat genetic transformation research in our laboratory, 77 wheat cultivars and lines with different LMW-GS allele compositions, 25 Aroona NILs, two F_2_ cross populations and 10 RILs. All materials used in this study were listed in Supplementary Table S4.

To estimate the performance of agronomic traits, yield and quality properties at different growing environments, CB037B and CB037C were planted at three locations of wheat production areas of north China (Beijing, Yinchuan and Xining) in 2015. Field trials were performed in randomized block design with three biological replicates (each plot with 30 m^2^). The cultivation and management were same as local field cultivation conditions.

### Grain developmental changes, agronomic trait and yield measurement

The grain dynamic changes from 4 to 24 DPA with two-day intervals were observed. The mature wheat plants from each plot were harvested and the main agronomic traits and grain yield were measured, including tiller number per plant, plant height, main ear length, effective ears per plant, ear grain number, thousand grain weight and grain yield (GY, kg/ha.).

### Glutenin extraction, SDS-PAGE and 2-DE

Seed glutenin extraction and SDS-PAGE were based on Mackie *et al*.^43^ and Yan *et al*.^44^. A 15 μL aliquot of the extract was loaded onto a SDS-PAGE gel. After electrophoresis with 12% gel at 15 mM for 2.5 h, the gel was stained for 30 min with Coomassie Brilliant Blue (CBB) R-250/G-250 (4:1) 10% (v/v) carbinol, 50% (v/v) acetic acid, and subsequently destained in distilled water.

For 2-DE, the first dimension was performed by an EttanTM IPG-phor II TM system (GE Healthcare, USA) using 18 cm strips (pH 6–11). The IEF rehydration solution was 7 M urea, 2 M thiourea and 4% CHAPS. The rehydrate condition was 30 V at 20 °C for 12 h while the IEF condition was 300 V for 1 h, 500 V for 1 h, 1000 V for 1 h, 3000 V for 1 h, and 8000 V to 80,000 V for 10 h. The second dimension was performed on a 12% acrylamide gradient. After electrophoresis, the 2-DE gels were stained within colloidal CBB R-250/G-250 (4:1) and analyzed by using ImageMaster™ 2-D platinum software version 5.0 (Amersham Bioscience, Swiss Institute of Bioinformatics, Geneva, Switzerland, 2003) based on Lv *et al*.^45^. Three biological replicates were performed.

### RP-UPLC

RP-UPLC was performed on an Agilent 1100 using a Zorbax 300SB-C18 column (300 Å pore size and 5 mm particle size) based on the recent report^46^.

### LC-MS/MS

The expected band on SDS-PAGE gel and 2-DE spots were excised and digested with trypsin according to Jin *et al*.^47^. The digested proteins (0.5 ml) were subject to LC-MS/MS identification by a Waters SYNAPT High Definition Mass Spectrometry™ mass spectrometer. Then the LC-MS/MS data were analyzed by software BioworksBrowser 3.3.

### DNA extraction and STS-PCR

Total genomic DNA was extracted from dry seeds according to McDonald *et al*.^48^ and An *et al*.^24^ with minor modifications. The molecular marker identification of *Glu-B3h* was performed using STS-PCR marker as previously report by Wang *et al*.^36^. The primer sets are SB8F: CCACCACAACAAACATTAA, and SB8R: GTGGTGGTTCTATACAACGA. The PCR cycling conditions were 94 °C for 4 min, followed by 35 cycles of 94 °C for 35 s, 60 °C for 35 s, 72 °C for 90 s, and a final extension at 72 °C for 8 min.

### Light microscopy and SEM observation

Transverse slices with approximately 1 mm thick were cut from the wheat grains during each of the four periods, and fixed, rinsed, dehydrated, infiltrated, and polymerized by the series of steps outlined in Arcalis *et al*.^49^ (2004). For light microscopy observation, sections with approximately 800 nm thick were cut, collected on mesh nickel grids, and stained with toluidine blue. The dynamic changes of endosperm structures from 4-day intervals after flowering during grain development were observed by SEM according to López-Merino *et al*.^50^ Collected developing grains were immediately fixed in the solution containing 44.5% ethanol, 1.85% methanal and 6% glacial acetic acid for 60 min followed by an overnight treatment at 4^o^ C, then transferred into 70% ethanol and stored at 4^o^ C prior to analysis. All samples were dehydrated sequentially through an ethanol concentration series (50%, 70%, 85%, 95% and 100% v/v) with 1 h incubation in each solution. After thoroughly dried, endosperm structures were observed by S-4800 FESEM machine (Hitachi, Japan).

### Gluten quality testing

Main quality parameters of mature grains from CB037B and CB037C from three locations were tested with three biological replications according to Zhen *et al*.^22^. Total protein content (%N 5.7, 14% moisture basis) was determined by nitrogen combustion analysis with a LECO (Model FP analyzer, St. Jopeph, MI) calibrated against EDTA. The extraction of GMP and content measurement were based on Xu *et al*.^51^ and Weegels *et al*.^52^. Separation of GMP by size exclusion-high performance liquid chromatography (SE-HPLC) were performed on a Phenomenex BIOSEPSEC 4000 column in acetonitrile buffer of 0.05% (v/v) triflouroacetic acid and 0.05% (v/v) acetonitrile with a running time of 10 m (2 ml/m flow rate) according to Rakszegia *et al*.^53^.

Mixograph parameters were measured with AACC54–40 A method by using 10 g electronic mixograph from America National manufacturing company. Extensograph parameters were tested based on He *et al*.^54^. For breadmaking quality testing, flour samples (100 g, 14% moisture base) and dry yeast were mixed at a ratio of 100:1 (w:w) together with water to develop the dough by using a laboratory Pin Mixer (National Manufacturing Company, Lincoln, NE). After that, the proofed dough was steamed in a steamer with boiling water for 15 min and cooled in the steamer for 15 min, finally took it out and cooled for 1 h at room temperature. Pan bread score included loaf volume (weighting 36), oven spring (10), appearance (10), texture (15), structure (15) and color (14) based on previous report^55^.

### AS-PCR

A pair of AS-PCR primer LB3F and LB3R was synthesized to amplify the full-length of *Glu-B3h* and part of its upstream and downstream sequence according to the results of tandem mass spectrometry and coding sequences of *GluB3-3* from GenBank (AC number EU369717), The primer sequences were LB3F: 5′-CATCACAAG CACAAGCATCAA-3′, LB3R: 5′-CATATCCATCGACTAAACAAA-3′, synthesized by Sangong Inc., China. PCR amplifications were performed in 50 μl reaction volumes containing 2.5 U La Taq polymerase (TaKaRa), 100 ng of template DNA, 25 μl of 2 × GC buffer I (MgCl_2_^ + ^plus), 0.4 mM dNTP, 0.5 μM of each primer, and added to 50 μl with double distilled H_2_O. The reactions were carried out in a PTC-100 (MJ Research, Watertown, MA, USA) thermocycler using the following protocol: 94 °C for 2 min, followed by 35 cycles of 94 °C for 45 s, 57 °C for 50 s and 72 °C for 1.5 min, a finally extension at 72 °C for 10 min.

### Molecular cloning, DNA sequencing and sequence alignment

PCR products were separated on 1.2% agarose gels in Tris-acetic acid-EDTA buffer and the expected fragments were purified from the gels using a Quick DNA extraction kit (Tiangen, Beijng, China). Subsequently, purified products were ligated into a PMD18–T Easy vector (TaKaRa, Dalian, China) and transformed into cells of *E. coli* strain DH5α according to Li *et al*^56^. DNA sequencing was performed with three clones by Sino Geno Max, Beijing, China. And the sequenced gene was blast in NCBI (http://www.ncbi.nlm.nih.gov/). Multiple sequence alignment of LMW-GS was performed by Bioedit 7.0.1.1.

### SNPs and InDels identification and secondary structure prediction

SNPs and InDels present in LMW glutenin genes were identified using Bioedit 7.0.1.1. Prediction of secondary structure of LMW-GS genes was conducted by PSIPRED server (http://bioinf.cs.ucl.ac.uk/psipred/).

### Phylogenetic tree construction

Clustal W program and MEGA 6.0 were used to construct phylogenetic tree and estimation of divergence times among LMW-GS genes. The Clustal W program was used to make a multiple alignment with homologous nucleotide sequences, the alignment file was converted to software MEGA 6.0 by the complete coding regions of LMW-GS genes with bootstrap values 1,000 replicates according to Johal *et al*.^57^. The divergence times of LMW-GS genes were estimated by using MEGA 6.0 with the evolution rate as 6.5 × 10^−9^ substitution/site year according to Allaby *et al*.^58^.

### Development and validation of SNP-based molecular markers for Glu-B3h

The specific primers for *Glu-B3h* gene were designed based on the SNP variations and used to develop molecular markers and then validated using different genotypes, Aroona NILs, F_2_ populations and RILs as shown in Supplementary Table S4. DNA extraction was as described above. PCR cycling conditions were 94 °C for 4 min, followed by 35 cycles of 94 °C for 35 s, 57 °C for 30 s, 72 °C for 90 s, and a final extension at 72 °C for 8 min. A total of 5–10 seeds for each cultivar, NIL and RIL, and 200–250 seeds from F_2_ populations were tested.

## Additional Information

**How to cite this article**: Wang, Y. *et al*. Low molecular weight glutenin subunit gene *Glu-B3h* confers superior dough strength and breadmaking quality in wheat (*Triticum aestivum* L.). *Sci. Rep.* **6**, 27182; doi: 10.1038/srep27182 (2016).

## Supplementary Material

Supplementary Information

## Figures and Tables

**Table 1:** Comparison of main quality parameters between CB037-B with Glu-B3h and CB037-C without Glu-B3h from three growing locations*.

Growing location	*Glu-B3h*	Total protein(%)	Wet glutenin(%)	GMP content**	Tolerance index (FU)	Development time (min)	Stability (min)	Max resistance (B.U.)	Extensibility (mm)	Loaf volume (ml^3^)	Loaf score
Yinchuan	*CB037-B* (+)	15.52 ± 0.1ab	58.45 ± 0.52a	8.75 ± 0.07a 1943.9 ± 36.2a	149 ± 2.86a	7.78 ± 0.08a	12.45 ± 0.11a	523 ± 5.51a	188 ± 1.17a	753 ± 6.17a	66 ± 0.56a
	*CB037-B* (−)	15.25 ± 0.08ab	56.52 ± 0.48a	8.16 ± 0.08b 1855.7 ± 38.3b	115 ± 2.74b	5.46 ± 0.06b	7.74 ± 0.09b	441b ± 4.12b	152 ± 1.11b	658 ± 6.74b	60 ± 0.45b
Beijing	*CB037-B* (+)	14.86 ± 0.08a	51.47 ± 0.42b	7.66 ± 0.08c 1885.9 ± 36.2c	145 ± 2.51a	7.42 ± 0.07a	11.85 ± 0.12a	483 ± 4.11c	173 ± 1.71a	746 ± 7.21a	64 ± 0.77a
	*CB037-B* (−)	14.92 ± 0.09a	50.82 ± 0.49b	6.50 ± 0.06d 1792.7 ± 36.2d	105 ± 1.78b	5.97 ± 0.06b	7.16 ± 0.07b	421b ± 4.32d	148 ± 1.53b	628 ± 6.12b	58 ± 0.21b
Xining	*CB037-B* (+)	15.87 ± 0.11b	62.25 ± 0.75c	8.86 ± 0.09a 1838.6 ± 31.7c	151 ± 2.36a	8.25 ± 0.09a	12.75 ± 0.11a	505 ± 5.01e	182 ± 1.73a	763 ± 7.44a	67 ± 0.63a
	*CB037-B* (−)	15.76 ± 0.12b	63.73 ± 0.88c	8.19 ± 0.07b 1784.6 ± 31.8d	118 ± 1.96b	5.46 ± 0.07b	7.96 ± 0.11b	457 ± 4.45f	159 ± 1.66b	605 ± 6.53b	52 ± 0.47b

^*^Different letters indicate significance level at *P* = 0.05.

^**^Upper values (%) and under values (Peak area, 1000 AU/S) were measured by the method of Weegels *et al*.^52^ and SE-HPLC, respectively.

**Table 2:** The positions of SNPs and InDels identified between *Glu-B3h* and other LMW-s genes.

LMW-GS	464–505	133	174	972	1110	1117	1172
EU369717	AACAACCAGTACTACCGCAACAACCACCATTTTCGCAGCAAC	A	C	A	T	T	G
20 other LMW-s genes	—	T	A	G	C	C	C

**Table 3:** The secondary structure prediction of the six deduced LMW-GS.

LMW-GS	Structure motifs	Contents (%)	Total	Dispersal in every region				
				N-terminal domain	Repetitive domain	C-ter domain I	C-ter domain II	C-ter domain III
AY542896	α-helix	2.72	1	–	–	–	–	1
	β-strand	1.09	2	–	–	1	–	1
AY831866	α-helix	5.98	3	–	–	2	–	1
	β-strand	1.09	2	–	–	–	1	1
AY724436	α-helix	–	–	–	–	–	–	–
	β-strand	1.32	2	–	–	–	–	2
FJ824789	α-helix	9.68	4	–	–	2	2	–
	β-strand	–	–	–	–	–	–	–
JX877832	α-helix	9.38	4	–	–	3	1	–
	β-strand	–	–	–	–	–	–	–
*Glu-B3h*	α-helix	9.95	4	–	–	3	1	–
	β-strand	0.54	1	–	–	–	–	1
