# Supplementary material for: Low molecular weight glutenin subunit gene Glu-B3h confers superior dough strength and breadmaking quality in wheat (Triticum aestivum L.)
Source: Sci Rep. 2016 Jun 7;6:27182. doi: 10.1038/srep27182 (PMC4895167; doi:10.1038/srep27182)
Supplement: Supplementary Information [file srep27182-s1.pdf]

Low molecular weight glutenin subunit gene *Glu-B3h* confers superior dough strength and breadmaking quality in wheat (*Triticum aestivum* L.)

Yaping Wang[1,4], Shoumin Zhen[1,4], Caixia Han[1], Xiaobin Lu[1], Xiaohui Li[1], Xianchun Xia[3], Zhonghu He[3] and Yueming Yan[*1,2]

Supplementary Data

Supplemental Table S1. Main agronomic traits and yield performance of CB037B and CB037C from three growing locations*

Location	Variety	Tiller number/plant	Plant height (cm)	Growing period (day)	Main ear length (cm)	Effective ears/plant	Ear grain number	Thousand kernel weight(g)	Grain yield (kg/ha.)
Yinchuan	CB037B	6.8±0.06a	65.8±0.52a	118.5±0.58	11.5±0.08a	6.6±0.05a	32.2±0.42a	37.7±0.46	4532±65a
	CB037C	6.9±0.07a	66.2±0.63a	119.2±0.62	12.1±0.09a	6.7±0.06a	31.8±0.45a	36.9±0.47	4538±68a
Beijing	CB037B	6.5±0.05b	61.7±0.42b	111.2±0.41	9.8±0.07b	5.6±0.02b	26.2±0.40b	36.7±0.44	4322±63b
	CB037C	6.2±0.04b	61.4±0.43b	111.4±0.65	9.6±0.06b	5.7±0.03b	25.8±0.41b	36.4±0.42	4329±65b
Xining	CB037B	6.6±0.06b	64.5±0.58c	115.6±0.47	10.8±0.05c	6.1±0.04c	28.2±0.44c	38.3±0.41	4488±67c
	CB037C	6.7±0.05c	64.8±0.53c	116.1±0.51	10.9±0.07c	5.9±0.03c	28.8±0.43c	38.6±0.45	4494±68c

*Different letters indicate significance level at P =0.05.

Supplemental Table S2. LC-MS/MS analyses of the *Glu-B3h* encoded LMW B-subunit separated by SDS-PAGE and 2-DE

Glu-B3h subunit		Peptide	Start	Stop*
SDS-PAGE		K.VFLQQQCSPVAM*PQSLAR.S	237	254
		R.TLPTMCNVNVSLYR.T	363	376
		R.TTTRVPFGVGTGVGGY.-	377	392
		R.VPFGVGTGVGGY.-	380	392
2-DE	Spot 1	R.TTTRVPFGVGTGVGGY.-	377	392
		R.VPFGVGTGVGGY.-	381	390
		R.TTTRVPFGVGTGVGG.-	377	391
	Spot 2	R.TTTRVPFGVGTGVGGY.-	377	392
		R.VPFGVGTGVGGY.-	381	392
		R.TTTRVPFGVGTGVGG.-	377	391
		R.VPFGVGTGVGG.-	381	391
	Spot 3	R.TTTRVPFGVGTGVGGY.-	377	392
		R.VPFGVGTGVGGY.-	380	392
		R.TTTRVPFGVGTGVGG.-	377	391
	Spot 4	R.TTTRVPFGVGTGVGGY.-	377	392
		R.VPFGVGTGVGGY.-	380	392
		R.TTTRVPFGVGTGVGG.-	377	391
		R.VPFGVGTGVGG.-	381	391

*The marched protein is Glu-B3-3 (AC number EU369717).

Supplemental Table S3. Estimation of divergence time (MYA) among 12 LMW-GS genes.

LMW-GS genes	Type	1	2	3	4	5	6	7	8	9	10	11	12
1. EU369717	LMW-s	—											
2. EU369700	LMW-s	2.46±0.46											
3. EU369722	LMW-s	2.62±0.54	2.54±0.54										
4. EU189088	LMW-s	2.54±0.46	0.31±0.15	2.46±0.46									
5. EU189095	LMW-s	2.23±0.46	2.08±0.46	2.31±0.46	2.00±0.46								
6. AB164416	LMW-s	2.77±0.54	0.54±0.23	2.62±0.54	0.23±0.15	2.00±0.46							
7. GQ892576	LMW-m	6.62±0.69	6.77±0.77	7.15±0.77	6.62±0.77	6.46±0.77	6.85±0.77						
8. GQ892588	LMW-m	6.54±0.77	6.54±0.77	7.15±0.77	6.46±0.77	6.31±0.77	6.62±0.77	4.77±0.69					
9. KC222115	LMW-m	6.77±0.77	6.77±0.77	7.38±0.77	6.62±0.77	6.54±0.77	6.85±0.77	4.92±0.69	0.62±0.23				
10. KC222119	LMW-m	6.46±0.77	6.46±0.77	7.08±0.77	6.31±0.77	6.23±0.77	6.54±0.77	4.62±0.62	0.31±0.15	0.54±0.23			
11. EU189087	LMW-i	11.92±1.00	11.92±1.00	12.31±1.00	12.00±1.00	11.31±1.00	12.23±1.00	13.15±1.00	12.15±1.00	12.62±1.00	12.31±1.00		
12.DQ307387	LMW-i	12.31±1.00	12.77±1.00	12.77±1.00	12.58±1.00	11.77±1.00	12.92±1.00	13.62±1.00	13.38±1.00	13.77±13.46	13.46±1.00	4.31±0.62	—

Supplemental Table S4. Materials used for developing and validating SNP-based molecular markers of *Glu-B3h* gene

No.	Materials	Origin	*Glu-A3*	*Glu-B3*	*Glu-D3*
	**Cultivars and lines:**				
1	CB037C	China	*Glu-A3a*	*null*	*Glu-D3d*
2	CB037B	China	*Glu-A3a*	*Glu-B3h*	*Glu-D3d*
3	CB037A	China	*Glu-A3a*	*Glu-B3h*	*Glu-D3d*
4	CS-1S^{l}(1B)	China	*Glu-A3a*	*Glu-B3a*	*Glu-D3a*
5	Ningchun 4	China	*Glu-A3a*	*Glu-B3a*	*Glu-D3a*
6	99G46	China	*Glu-A3f*	*Glu-B3j*	*Glu-D3c*
7	Zhongyu 415	China	*Glu-A3c*	*Glu-B3d*	*Glu-D3c*
8	Demai 3	China	*Glu-A3c*	*Glu-B3i*	*Glu-D3b*
9	Fengmai 27	China	*Glu-A3c*	*Glu-B3f*	*Glu-D3a*
10	Guanfeng 2	China	*Glu-A3c*	*Glu-B3b*	*Glu-D3a*
11	Lumai 23	China	*Glu-A3c*	*Glu-B3d*	*Glu-D3c*
12	Neixiang 188	China	*Glu-A3a*	*Glu-B3j*	*Glu-D3a*
13	Shan 229	China	*Glu-A3c*	*Glu-B3j*	*Glu-D3b*
14	Wanmai 33	China	*Glu-A3d*	*Glu-B3g*	*Glu-D3a*
15	Yan 239	China	*Glu-A3c*	*Glu-B3j*	*Glu-D3b*
16	Yangmai 158	China	*Glu-A3c*	*Glu-B3g*	*Glu-D3c*
17	Yumai 54	China	*Glu-A3c*	*Glu-B3d*	*Glu-D3c*
18	Yumai 63	China	*Glu-A3c*	*Glu-B3d*	*Glu-D3c*
19	Yumai 69	China	*Glu-A3c*	*Glu-B3d*	*Glu-D3a*
20	Zhongyou 9507	China	*Glu-A3d*	*Glu-B3b*	*Glu-D3c*
21	Zhongyou 9701	China	*Glu-A3d*	*Glu-B3d*	*Glu-D3c*
22	Huaimai 16	China	*Glu-A3f*	*Glu-B3h*	*Glu-D3c*
23	Jing 411	China	*Glu-A3c*	*Glu-B3h*	*Glu-D3c*
24	CA9722	China	*Glu-A3c*	*Glu-B3h*	*Glu-D3c*
25	CA9641	China	*Glu-A3d*	*Glu-B3h*	*Glu-D3c*
26	WAWHT3060	China	*Glu-A3f*	*Glu-B3h*	*Glu-D3c*
27	WAWHT3122	China	*Glu-A3c*	*Glu-B3h*	*Glu-D3a*
28	Petrel	France	*Glu-A3d*	*Glu-B3h*	*Glu-D3c*
29	hartog	France	*Glu-A3d*	*Glu-B3h*	*Glu-D3c*
30	Stiletto	Australia	*Glu-A3c*	*Glu-B3h*	*Glu-D3c*
31	Spear	Australia	*Glu-A3e*	*Glu-B3h*	*Glu-D3c*
32	Trident	Australia	*Glu-A3e*	*Glu-B3h*	*Glu-D3c*

33	Wilgoyne	Australia	*Glu-A3d*	*Glu-B3h*	*Glu-D3b*
34	Aca 303	Argentina	*Glu-A3f*	*Glu-B3h*	*Glu-D3c*
35	Klein Capricornio	Argentina	*Glu-A3c*	*Glu-B3h*	*Glu-D3b*
36	Klein Chaja	Argentina	*Glu-A3c*	*Glu-B3h*	*Glu-D3b*
37	Klein Flecha	Argentina	*Glu-A3c*	*Glu-B3h*	*Glu-D3b*
38	ProINTA Redomon	Argentina	*Glu-A3c*	*Glu-B3h*	*Glu-D3c*
39	Nidera Baguette 10	Argentina	*Glu-A3d*	*Glu-B3g*	*Glu-D3c*
40	Nidera Baguette 20	Argentina	*Glu-A3f*	*Glu-B3g*	*Glu-D3c*
41	Thomas Nevado	Argentina	*Glu-A3c*	*Glu-B3j*	*Glu-D3b*
42	Gabo	France	*Glu-A3b*	*Glu-B3b*	*Glu-D3b*
43	Orca	France	*Glu-A3d*	*Glu-B3d*	*Glu-D3c*
44	KAUZ//ALTAR 84/AOS/3/MILAN/KAUZ/4/HUITES	CIMMYT	*Glu-A3b*	*Glu-B3b*	*Glu-D3a*
45	MILAN/S87230//BABAX	CIMMYT	*Glu-A3b*	*Glu-B3i*	*Glu-D3a*
46	ALTAR 84/AEGILOPS SQUARROSA (TAUS)//OPATA	CIMMYT	*Glu-A3b*	*Glu-B3i*	*Glu-D3a*
47	Festin	France	*Glu-A3f*	*Glu-B3b*	*Glu-D3c*
48	ATTILA/3*BCN//BAV92/3/PASTOR	CIMMYT	*Glu-A3c*	*Glu-B3j*	*Glu-D3b*
49	Apollo	France	*Glu-A3d*	*Glu-B3j*	*Glu-D3c*
50	Pepital	France	*Glu-A3f*	*Glu-B3d*	*Glu-D3c*
51	Salmone	France	*Glu-A3c*	*Glu-B3c*	*Glu-D3c*
52	Brimstone	France	*Glu-A3c*	*Glu-B3g*	*Glu-D3d*
53	Cappelle-Desprez	France	*Glu-A3d*	*Glu-B3g*	*Glu-D3c*
54	Magali Blondeau	France	*Glu-A3e*	*Glu-B3f*	*Glu-D3b*
55	Chinese Spring	China	*Glu-A3a*	*Glu-B3a*	*Glu-D3a*
56	ACHTAR*3//KANZ/KS85-8-5	CIMMYT	*Glu-A3b*	*Glu-B3g*	*Glu-D3a*
57	Manital	France	*Glu-A3c*	*Glu-B3b*	*Glu-D3a*
58	Oberkulmer	Switzerland	*Glu-A3h*	*Glu-B3d*	*Glu-D3a*
59	Fuggers	Primitive spelt	*Glu-A3h*	*Glu-B3d*	*Glu-D3a*
60	Babenhauser	Germany	*Glu-A3h*	*Glu-B3d*	*Glu-D3a*
61	Altgold	Switzerland	*Glu-A3h*	*Glu-B3d*	*Glu-D3a*
62	Ostro	Switzerland	*Glu-A3h*	*Glu-B3d*	*Glu-D3a*
63	Hubel	Switzerland	*Glu-A3a*	*Glu-B3b*	*Glu-D3a*
64	Rouguin	Belgium	*Glu-A3a*	*Glu-B3d*	*Glu-D3a*
65	Schwabenkorn	Germany	*Glu-A3h*	*Glu-B3d*	*Glu-D3a*
66	Hercule	Belgium	*Glu-A3h*	*Glu-B3c*	*Glu-D3a*
67	Franckenkorn	Germany	*Glu-A3a*	*Glu-B3d*	*Glu-D3a*

68	Renval	Belgium	*Glu-A3a*	*Glu-B3d*	*Glu-D3a*
69	Waggershauser Hohenheimer	Germany	*Glu-A3a*	*Glu-B3b*	*Glu-D3a*
70	Buck Brasil	Argentina	*Glu-A3f*	*Glu-B3g*	*Glu-D3d*
71	Buck Pingo	Argentina	*Glu-A3f*	*Glu-B3i*	*Glu-D3c*
72	Angas	Australia	*Glu-A3c*	*Glu-B3g*	*Glu-D3c*
73	Avocent	Australia	*Glu-A3c*	*Glu-B3b*	*Glu-D3b*
74	Grebe	Australia	*Glu-A3c*	*Glu-B3j*	*Glu-D3b*
75	Halberd	Australia	*Glu-A3e*	*Glu-B3c*	*Glu-D3c*
76	Millewa	Australia	*Glu-A3c*	*Glu-B3g*	*Glu-D3b*
77	Bluesky	Canada	*Glu-A3g*	*Glu-B3g*	*Glu-D3c*
78	Glenlea	Canada	*Glu-A3g*	*Glu-B3g*	*Glu-D3c*
79	Marquis	Canada	*Glu-A3e*	*Glu-B3b*	*Glu-D3a*
**F_2 populations:**					
80	F_2-1	CB037B × Ningchun 4			
81	F_2-2	CS-1S^l(1B) × CB037B			
**NILs:**					
82	Aroona	Australia	*Glu-A3c*	*Glu-B3b*	*Glu-D3c*
83	Aril2-4	Australia	*Glu-A3c*	*Glu-B3b*	*Glu-D3c*
84	Aril3-2	Australia	*Glu-A3c*	*Glu-B3b*	*Glu-D3c*
85	Aril5-2	Australia	*Glu-A3c*	*Glu-B3b*	*Glu-D3c*
86	Aril7-1	Australia	*Glu-A3c*	*Glu-B3b*	*Glu-D3c*
87	Aril9-3	Australia	*Glu-A3c*	*Glu-B3b*	*Glu-D3c*
88	Aril10-1	Australia	*Glu-A3c*	*Glu-B3b*	*Glu-D3c*
89	Aril12-3	Australia	*Glu-A3c*	*Glu-B3b*	*Glu-D3c*
90	Aril13-3	Australia	*Glu-A3c*	*Glu-B3b*	*Glu-D3c*
91	Aril14-3	Australia	*Glu-A3c*	*Glu-B3b*	*Glu-D3c*
92	Aril16-1	Australia	*Glu-A3b*	*Glu-B3b*	*Glu-D3c*
93	Aril18-5	Australia	*Glu-A3d*	*Glu-B3b*	*Glu-D3c*
94	Aril19-2	Australia	*Glu-A3e*	*Glu-B3b*	*Glu-D3c*
95	Aril20-1	Australia	*Glu-A3f*	*Glu-B3b*	*Glu-D3c*
96	Aril21-2	Australia	*Glu-A3c*	*Glu-B3a*	*Glu-D3c*
97	Aril23-4	Australia	*Glu-A3c*	*Glu-B3c*	*Glu-D3c*
98	Aril24-3	Australia	*Glu-A3c*	*Glu-B3d*	*Glu-D3c*
99	Aril26-1	Australia	*Glu-A3c*	*Glu-B3f*	*Glu-D3c*
100	Aril27-6	Australia	*Glu-A3c*	*Glu-B3g*	*Glu-D3c*
101	Aril29-4	Australia	*Glu-A3c*	*Glu-B3i*	*Glu-D3c*
102	Aril28-4	Australia	*Glu-A3c*	*Glu-B3h*	*Glu-D3c*
103	Aril30-1	Australia	*Glu-A3c*	*Glu-B3b*	*Glu-D3a*

104	Aril36-2	Australia	*Glu-A3c*	*Glu-B3b*	*Glu-D3b*
105	Aril33-1	Australia	*Glu-A3c*	*Glu-B3b*	*Glu-D3d*
106	Aril35-1	Australia	*Glu-A3c*	*Glu-B3b*	*Glu-D3f*
	**RILs:**				
107	RIL-1 (CB037B × Ningchun 4)	China	*Glu-A3a*	*Glu-B3h*	*Glu-D3a*
108	RIL-2 (CB037B × Ningchun 4)	China	*Glu-A3a*	*Glu-B3h*	*Glu-D3d*
109	RIL-3 (CB037B × Ningchun 4)	China	*Glu-A3a*	*Glu-B3a*	*Glu-D3d*
110	RIL-4 (CB037B × Ningchun 4)	China	*Glu-A3a*	*Glu-B3a*	*Glu-D3a*
111	RIL-5 (CB037B × Ningchun 4)	China	*Glu-A3a*	*Glu-B3a*	*Glu-D3d*
112	RIL-6 (CS-1S^l(1B) × CB037B)	China	*Glu-A3a*	*Glu-B3h*	*Glu-D3d*
113	RIL-7 (CS-1S^l(1B) × CB037B)	China	*Glu-A3a*	*Glu-B3h*	*Glu-D3a*
114	RIL-8 (CS-1S^l(1B) × CB037B)	China	*Glu-A3a*	*Glu-B3a*	*Glu-D3d*
115	RIL-9 (CS-1S^l(1B) × CB037B)	China	*Glu-A3a*	*Glu-B3a*	*Glu-D3a*
116	RIL-10 (CS-1S^l(1B) × CB037B)	China	*Glu-A3a*	*Glu-B3a*	*Glu-D3d*

Supplemental Fig. S1. Plant, seeds and spikelet morphology of CB037B and CB037C in Yinchuan.

Supplemental Fig. S2. Identification of *Glu-B3h* by STS-PCR markers. 1. CB037C, 2. CB037B, 3. Aroona-B3a, 4. Aroona-B3b, 5. Aroona-B3d, 6. Aroona-B3f, 7. Marker (8000bp, 7000bp, 6000bp, 5000bp, 4000bp, 3000bp, 2000bp, 1000bp), 8. Aroona-B3g, 9. Aroona-B3h, 10. Aroona-B3i, 11. CS.

Supplemental Fig. S3. Sequence alignment of STS-PCR marker products of CB037B and Aroon-B3h, *Glu-B3h* and its CDS. EU369717 *B3h* is the full sequence, EU369717 CDS is the coding area, CB037B and *Glu-B3h* is the band we cloned with the marker (SB8F, SB8R) from CB037B and Aroon-B3h.

Supplemental Fig. S4. Agarose gel electrophoresis separation of amplified products from genomic DNA of CB037B. With the AS-PCR primer, a single band was cloned in CB037B . Lane1: PCR amplified products and Lane2: 1Kb DNA marker.

Supplemental Fig. S5. Multiple alignment of the deduced amino acid sequences of Glu-B3h and other 26 LMW-s glutenin genes. These genes including GenBank number EU369711, EU369712, EU369713, EU369714, EU369715, EU369716, EU369718, AB062853, AB119006, AB164415, DQ357057, EF437420, EF437421, EF437422, EF437423, EF437425, EF437426, FJ755309, FJ824787, FJ824788, FJ824789, JF339167, JX877832, JX878086, JX878206 and Y18159. Signal represents signal peptide, N-terminal sequence (I), repetitive domain (II) and three sub-regions of C-terminal domain (III, IV, V) were indicated, respectively. The first amino acid residue of the mature proteins and cysteine residues were highlighted by red box and red shading, respectively. Deletions were indicated by dashes.

Supplemental Fig. S6. SDS-PAGE of glutenin subunits. (a) Glutenin subunits from different wheat cultivar: 1. CB037C. 2-17 (Table S1: 42-57). 18. Petrel. 19. Hartog. 20. CB037B. **(b)** Glutenin subunits from F_2 populations of CB037B × Ningchun 4 (3-17). 1. CB037B. 2. CB037C. **(c)** Glutenin subunits from Aroona NILs 2-8 corresponding to 96-102 in Table S4. 1. CB037C. 9. CB037B.

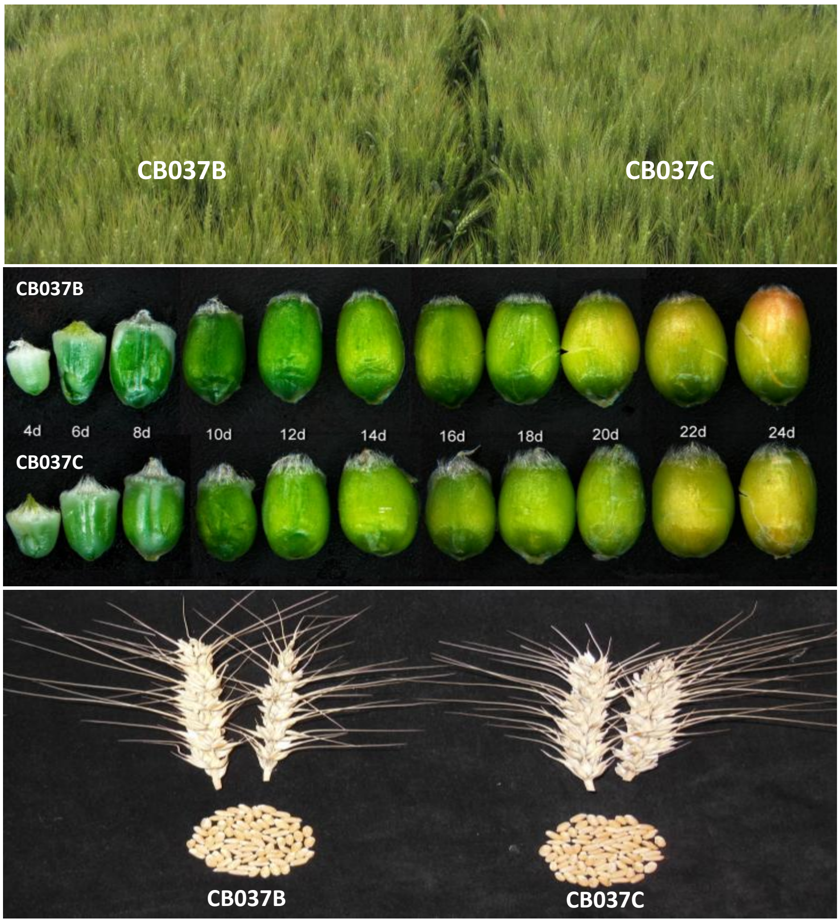
CB037B
CB037C
CB037B
4d
6d
8d
10d
12d
14d
16d
18d
20d
22d
24d
CB037C
CB037B
CB037C

Fig. S1

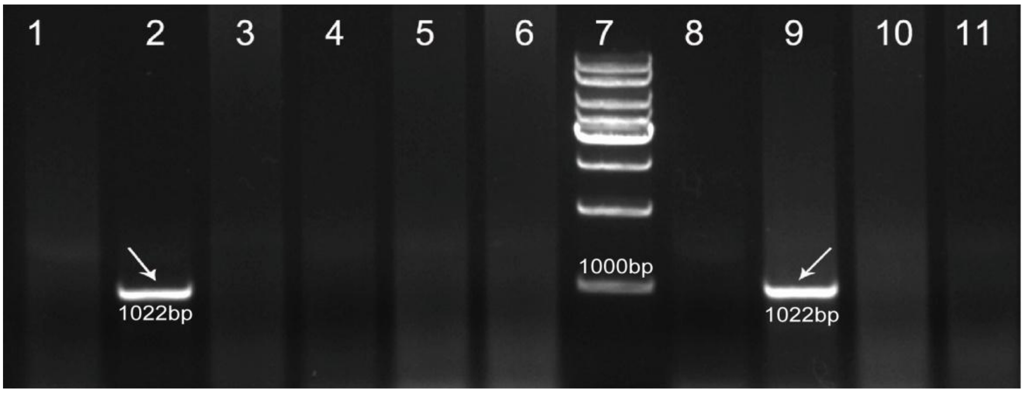

Fig. S2

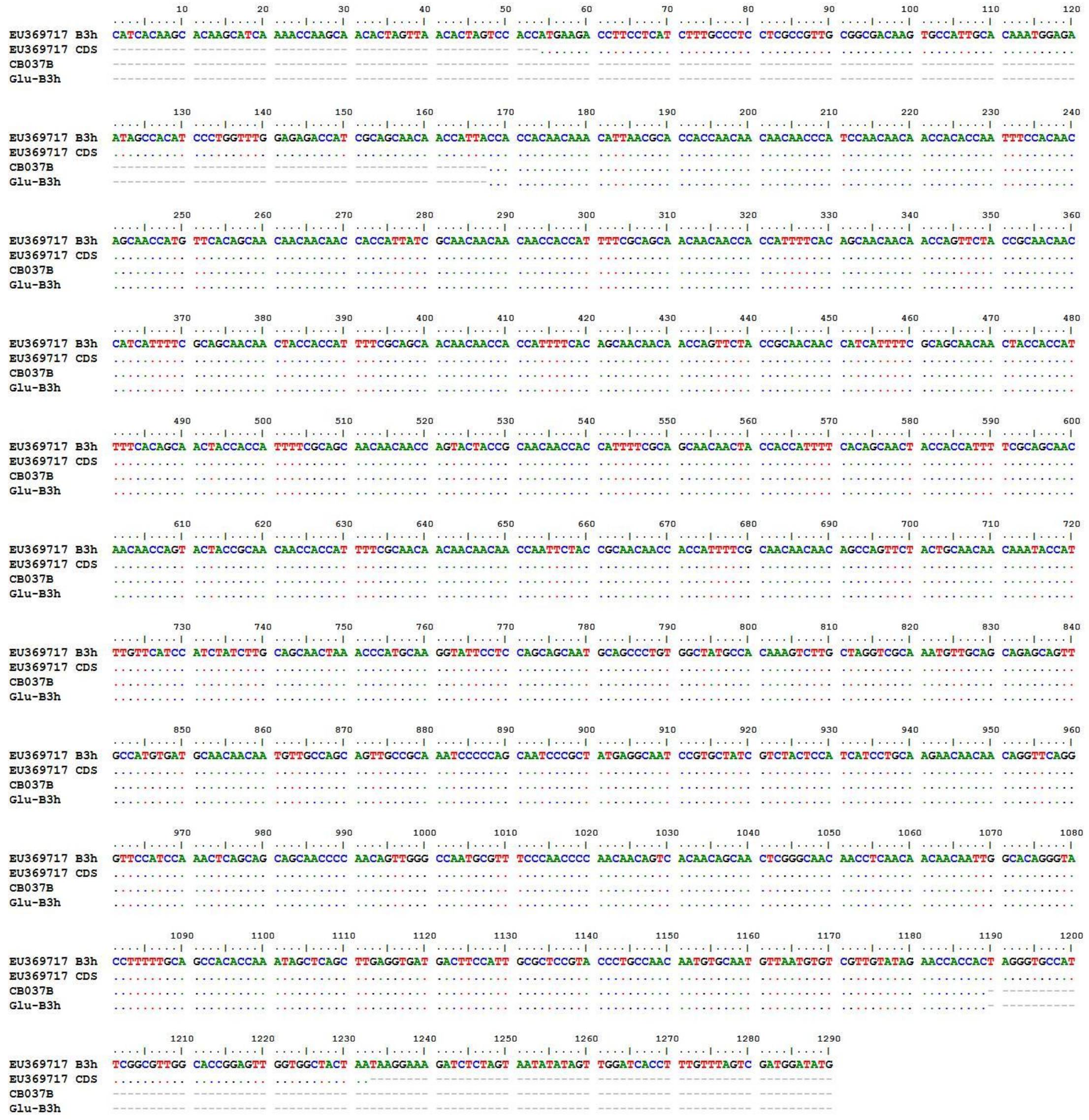

Fig. S3

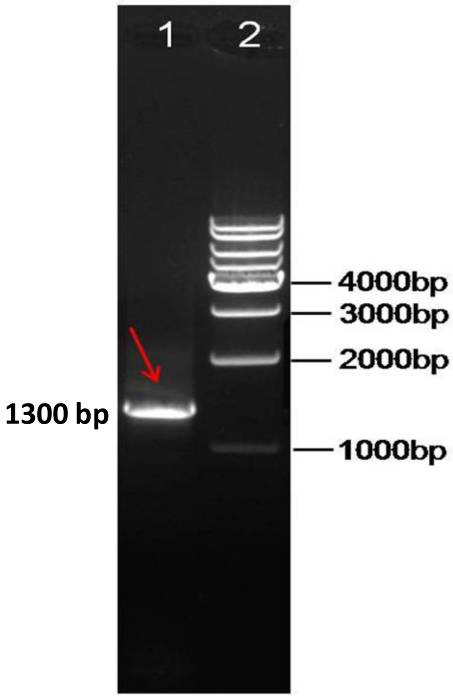

Fig. S4

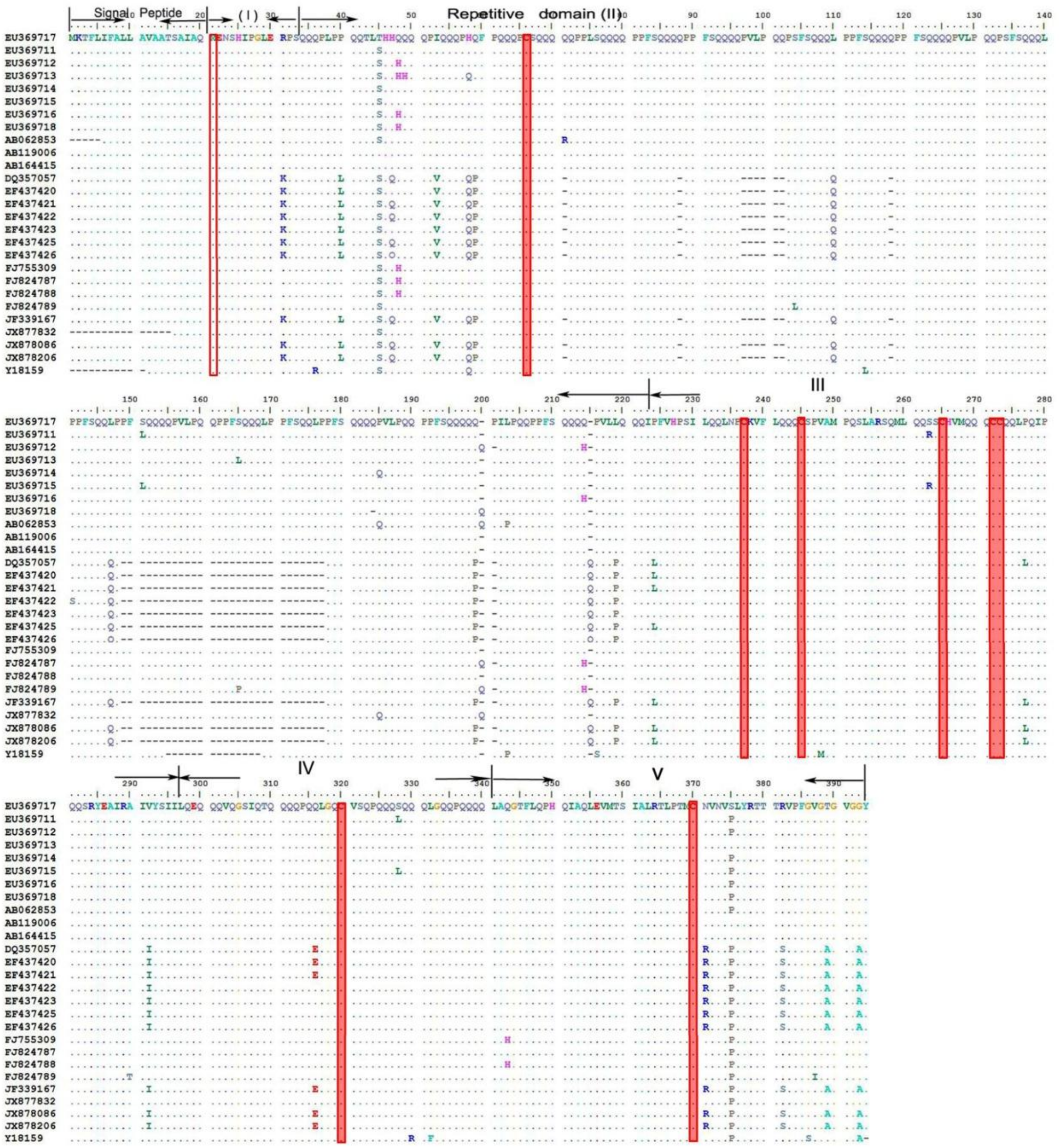

Fig. S5

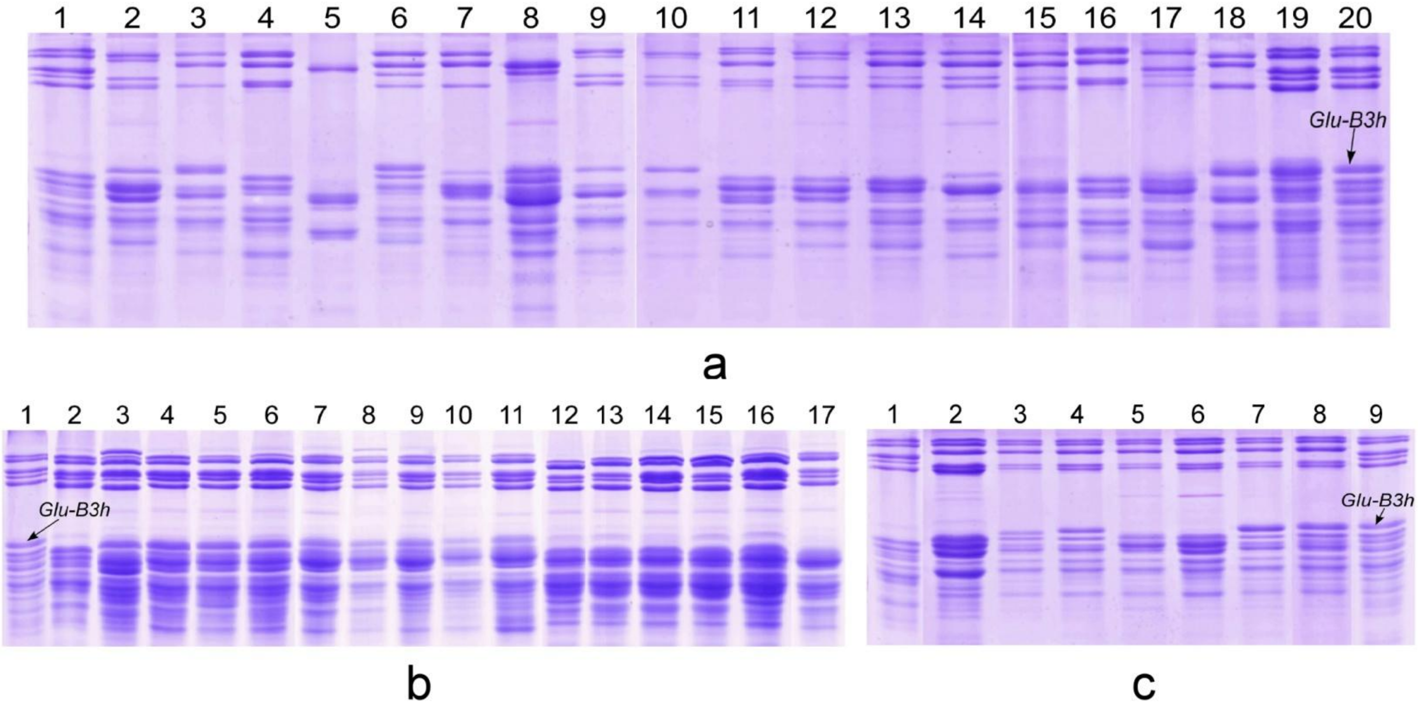
1 2 3 4 5 6 7 8 9 10 11 12 13 14 15 16 17 18 19 20
Glu-B3h
a
1 2 3 4 5 6 7 8 9 10 11 12 13 14 15 16 17
Glu-B3h
b
1 2 3 4 5 6 7 8 9
Glu-B3h
c

Fig. S6
